# An Exploratory Analysis of Tumor Site- and Sex-Specific Associations of SNPs of LncRNA CCAT1, CCAT2, H19, HOTAIR, and PTCSC3 in Colorectal Lesions: A Hungarian Case–Control Study

**DOI:** 10.3390/biomedicines13123058

**Published:** 2025-12-11

**Authors:** Krisztina Varajti, Andrea Vereczkei, Márk Kovács-Valasek, Afshin Zand, Tímea Varjas, István Kiss

**Affiliations:** 1 Department of Public Health Medicine, Medical School, University of Pécs, 7624 Pécs, Hungary; timea.varjas@aok.pte.hu (T.V.); istvan.kiss@aok.pte.hu (I.K.); 2 Department of Molecular Biology, Institute of Biochemistry and Molecular Biology, Semmelweis University, 1094 Budapest, Hungary; vereczkei.andrea@semmelweis.hu; 3 Pannonpharma Ltd., 7720 Pécsvárad, Hungary; kovacs.m@pannonpharma.hu

**Keywords:** colorectal cancer, lncRNA, CCAT1, CCAT2, HOTAIR, H19, PTCSC3, case-control study, single nucleotide polymorphism, Hungarian population

## Abstract

**Background:** Colorectal cancer is a major public health burden in Hungary, with one of the highest incidence and mortality rates in Europe. Long non-coding RNAs (lncRNAs) have emerged as key regulators in tumorigenesis, but population-specific genetic associations remain understudied. This study aimed to investigate whether single-nucleotide polymorphisms (SNPs) in lncRNA genes are associated with colorectal cancer susceptibility, with attention to tumor site- and sex-specific effects. **Methods:** We conducted an exploratory case–control study involving 91 Hungarian participants (38 patients with colorectal lesions and 53 controls). Genotyping of six SNPs located in HOTAIR, PTCSC3, H19, CCAT1, and CCAT2 was performed using TaqMan-based qPCR. Associations were tested using allele frequency analysis, different genotype models (dominant, recessive, additive), and binary logistic regression, including stratified analyses by tumor subtype and sex. **Results:** While no significant associations were found in the unadjusted overall case–control comparisons, logistic regression including sex revealed that HOTAIR rs12826786 and rs7958904 were significantly associated with a reduced risk of colorectal lesions, particularly in females (*p* = 0.022 and *p* = 0.043). Analyses by tumor localization revealed that H19 rs2839698 and PTCSC3 rs944289 were more frequent in colon than in rectal tumors (*p* = 0.017 and *p* = 0.035) and were associated with a reduced risk of rectal tumors (OR = 0.18 and OR = 0.20), suggesting that these variants may influence tumor site rather than overall susceptibility. None of the results remained significant after Bonferroni correction. **Conclusions:** Our findings suggest that these selected lncRNA-related SNPs may contribute to colorectal cancer risk in a sex- and site-specific manner. These preliminary results warrant further validation in larger, independent cohorts and functional studies.

## 1. Introduction

Colorectal cancer (CRC) is a significant public health concern worldwide. It is the third-most commonly diagnosed malignancy and the second leading cause of cancer-related deaths globally with significant geographic variation in incidence and mortality rates [1]. Developed countries in Europe, North America, and Australia report particularly high rates, reflecting the combined impact of lifestyle factors, screening programs, and healthcare systems. In Europe, Hungary has one of the highest incidence and mortality rates. According to the Global Cancer Observatory, for 2022 the age-standardized incidence rate (ASR) is approximately 62.4 and 30.4 per 100,000 for men and women, respectively, and the mortality rate reached 20.2 per 100,000 overall, which places Hungary among the top countries globally [1]. For the same year, a total of 11,020 new cases were diagnosed according to the Hungarian National Cancer Registry [2]. The high rates may reflect late-stage diagnosis, which can occur due to inadequate screening programs and public awareness. These data underline the urgent need for improved strategies for prevention and early detection in the Hungarian population. Recently, increasing focus has been placed on the contribution of genetic and epigenetic factors, including long non-coding RNAs (lncRNAs), to CRC pathogenesis. Long non-coding RNAs—a class of RNA molecules longer than 200 nucleotides that do not code for proteins—have emerged in the past few years as key regulators of gene expression at multiple levels, including chromatin remodeling [3,4], transcription, and post-transcriptional modifications [5,6,7]. Dysregulation of lncRNAs has been linked to tumor initiation, progression, metastasis, and chemoresistance in several studies [8,9,10,11,12,13]. Importantly, lncRNA genes are often affected by single-nucleotide polymorphisms (SNPs), which may have an impact on their expression or function, thereby modifying individual susceptibility to cancer [14,15,16,17,18,19]. Among the lncRNAs connected to CRC, several have been intensively studied [20,21,22]. Colon cancer-associated transcript 1 (CCAT1) and colon cancer-associated transcript 2 (CCAT2), both located on chromosome 8q24.21, promote tumorigenesis by enhancing *MYC* expression and activating WNT/β-catenin signaling with elevated expression in tumor tissues [23,24]. H19 is an imprinted lncRNA with increased expression reported in CRC in both serum samples [25] and tumor tissue [26,27], contributing to invasion, metastasis, and poor prognosis. HOX transcript antisense RNA (HOTAIR) transcribed from the *HOXC* locus, drives epigenetic silencing of tumor suppressor genes [28] and is associated with advanced disease and poor survival [29,30,31,32], based on tumor tissue experiments. Conversely, while papillary thyroid carcinoma susceptibility candidate 3 (PTCSC3) is well established as a tumor-suppressive lncRNA, partly through the inhibition of oncogenic signaling pathways, it is consistently downregulated in thyroid carcinoma and has been reported in several other digestive malignancies [33,34,35,36,37]. However, its potential role in CRC remains less defined, with current evidence largely limited to associative observations rather than mechanistic validation.

These molecules also hold considerable promise as diagnostic and prognostic biomarkers, reflecting their altered expression in serum and their high stability in tissue models. Notably, several lncRNAs can be detected in circulation, for example, CCAT1 and CCAT2 levels in serum extracellular vesicles or exosomes closely parallel their abundance in tumor tissue, and their elevated concentrations in both compartments are associated with diagnostic and prognostic significance [38,39].

Given the high CRC burden in Hungary and the growing evidence of lncRNA-related genetic susceptibility, this study aimed to investigate particular SNPs within these lncRNA genes in a Hungarian case–control cohort. These SNPs were chosen a priori based on previously published reports (Table 1) to explore whether there are differences in genotype frequencies between Hungarian patients with colorectal lesions and non-cancer controls. Based on previous evidence implicating these loci in colorectal carcinogenesis, we hypothesized that the selected SNPs would be associated with the risk of colorectal neoplasms in our study population.

## 2. Materials and Methods

### 2.1. Patients and Samples

We recruited Hungarian patients from the Department of Surgery of the University of Pécs Medical School starting from September 2022. Informed consent was received from all subjects. A total of 4 mL whole blood was collected in ethylenediaminetetraacetic acid (EDTA) tubes (Greiner Bio-One International GmbH, Kremsmünster, Austria) and stored at −20 °C until DNA isolation. Patients with confirmed colon or rectal cancer or lesions were enrolled in the case group, while individuals without cancer were enrolled as controls. Exclusion criterion for cases included clinically proven presence of any other neoplasm other than colorectal lesions; for controls, exclusion was the presence of any cancer. Both groups consisted of Hungarian individuals aged 28 to 93 years, with no restriction on sex (male-to-female ratios were 1:1.24 in cases and 1:1.65 in controls). The study was approved by the Scientific and Research Ethics Committee of Medical Research Council (ETT-TUKEB) under the registration number 39065-5/2022/EÜIG and was conducted according to the Declaration of Helsinki. Until April 2025, 93 blood samples have been collected and selected for DNA isolation and inclusion in this study.

### 2.2. DNA Isolation

DNA isolation was performed using High Pure PCR Template Preparation Kit (Roche Diagnostics GmbH, Mannheim, Germany). Isolation was performed from 200 µL whole blood using the working solutions for the preparation and centrifugation steps according to the manufacturer’s protocol. The purity of the isolated DNA was checked by spectrophotometry with MaestroGen Nano spectrophotometer (MaestroGen Inc., Hsinchu City, Taiwan) using the OD ratio of A260/A280 nm.

### 2.3. Genotyping with qPCR

We performed qPCR-based genotyping with sequence-specific TaqMan^®^ assays (Thermo Fisher Scientific, Waltham, MA, USA) on QuantStudio 12K Flex Real-Time PCR System (Thermo Fisher Scientific, Waltham, MA, USA). Assay IDs: CCAT1 rs6708563: C_189159697_10, CCAT2 rs6983267: C__29086771_20, H19 rs2839698: C___2603701_10, HOTAIR rs7958904: C___2104252_20, HOTAIR rs12826786: C__31185830_10, PTCSC3 rs944289: C___1444137_10. TaqPath™ ProAmp™ mastermix (Thermo Fisher Scientific, Waltham, MA, USA) was used. Thermal profile for all runs were as follows: hold stage: 10:00 min at 95 °C; PCR stage: 00:15 min at 95 °C then 01:00 min at 60 °C; post-read stage: 01:00 min at 60 °C. A total of 43 cycles were used. Automatic genotype calling was performed by the QuantStudio 12K Flex System software with a successful call rate higher than 95% for all assays. Samples with failed calls were excluded from further analyses. All PCR runs were performed in accordance with the given standards and operational procedures, including two negative controls per run, which remained ‘undetermined’ after PCR.

### 2.4. Statistical Analyses

All statistical analyses were performed using IBM SPSS Statistics version 27 (IBM Corporation, Armonk, NY, USA). Descriptive statistics were calculated for demographic and clinical variables, including means, standard deviations, and frequency distributions. Group differences in age and sex were assessed using χ^2^ tests. SNPs were analyzed under additive, dominant, and recessive genetic models. Genotype distributions were tested for deviation from Hardy–Weinberg equilibrium (HWE) using χ^2^ tests. Allelic and genotype associations with disease status were examined using χ^2^ tests, Fisher’s exact test, and binary logistic regression. Subgroup analyses were conducted by stratifying cases according to tumor location (colon vs. rectum) and sex. To evaluate linear trends across genotype categories, linear-by-linear association tests were used where appropriate. Odds ratios (ORs) and 95% confidence intervals (CIs) were reported for effect estimates. A *p*-value < 0.05 was considered nominally significant. Multiple testing correction was applied using the Bonferroni method. To evaluate the statistical power of our study, we performed a post hoc power analysis based on the observed average minor allele frequencies (MAF = 0.38) and the total sample size, using χ^2^-based effect sizes (Cohen’s w).

## 3. Results

### 3.1. Sample Characteristics of the Study’s Population

Two samples consistently failed to yield a signal in all PCR runs and were therefore excluded from the analysis. Consequently, 91 samples were included in the final dataset, comprising 38 cases and 53 controls (Table 2). Genotype data are represented in Supplementary Table S1. The case group consisted of individuals diagnosed with colorectal or benign colon tumors, while the control group included cancer-free participants. The distribution of sex did not differ significantly between the two groups (χ^2^ = 0.21, *p* = 0.650). The median age was 71.5 years (IQR: 58.0–75.25) in the case group and 60.0 years (IQR: 52.8–69.8) in the control group. Among the cases, colon cancer was the most common diagnosis (63.1%), followed by rectal cancer (31.6%) and benign colon tumors (5.3%) (Table 3). Genotype counts for all models for each SNP are represented in Table 4. The MAF observed in our Hungarian controls were generally comparable to those reported for the 1000 Genomes European and gnomAD non-Finnish European population, with only modest differences for most SNPs, indicating that our findings are generalizable to European populations. The largest divergence was observed for CCAT1 (16.0% vs. 30.7% vs. 32.5%), which permits cautious interpretation and independent replication (Table 4).

### 3.2. No Significant Deviation from Hardy–Weinberg Equilibrium (HWE) in the Study’s Control Population

Hardy–Weinberg equilibrium (HWE) was assessed for each SNP in the control group (*n* = 53) according to Wigginton et al. [40]. Observed genotype counts (homozygous for the major allele, heterozygous, homozygous for the minor allele) were obtained directly from the dataset (Supplementary Table S2). No significant deviation from HWE was found for any of the SNPs (Table 5).

### 3.3. Genotype Distributions and Allele Frequencies Did Not Differ Significantly Between Cases and Controls

We analyzed the genotype distribution of the six investigated SNPs associated with lncRNAs (H19 rs2839698, CCAT1 rs6708563, CCAT2 rs6983267, HOTAIR rs7958904, HOTAIR rs12826786, PTCSC3 rs944289) in case and control groups under three genetic models: additive, dominant, and recessive (see Supplementary Table S3). No statistically significant differences were observed between cases and controls across any of the SNPs. Suggestive associations emerged under dominant models for HOTAIR rs7958904 and rs12826786 (*p* = 0.075 and *p* = 0.097, respectively). Given the relatively small sample size, Fisher’s exact test was additionally applied for the dominant and recessive models, but this analysis also yielded no statistically significant associations (Supplementary Table S4). The results are not adjusted for multiple testing and should therefore be interpreted with caution.

No statistically significant differences were observed in allele distributions between cases and controls for any of the six SNPs (Supplementary Table S5). The lowest *p*-value was found for CCAT1 rs6708563 (*p* = 0.089), as there was a higher frequency of the T allele in colorectal cancer cases compared to controls (26.3% vs. 16.0%), although this difference did not reach statistical significance.

### 3.4. Sex-Specific Associations of HOTAIR Polymorphisms with Colorectal Lesion Risk

Significant associations were observed for the HOTAIR polymorphisms in sex interaction models using logistic regression. In female participants, HOTAIR rs12826786 was significantly associated with a reduced risk of disease, as the presence of the minor allele (T) was associated with decreased odds of being a case (*p* = 0.022, OR = 0.29, 95% CI: 0.10–0.84, under the additive model). The association remained significant under the dominant model as well (*p* = 0.033, OR = 0.29, 95% CI: 0.09–0.90). Also, for female patients, carrying at least one C allele at HOTAIR rs7958904 was found to be associated with a significant protective effect (*p* = 0.043, OR = 0.31, 95% CI: 0.10–0.96, under the dominant model). This suggests that the protective effect of the minor alleles may be present only in females.

In addition to these findings, several SNPs demonstrated suggestive associations. Under the additive model including sex and SNP interaction, HOTAIR rs12826786 showed a non-significant increased odds of disease in men compared with women (*p* = 0.057, OR = 4.01, 95% CI: 0.96–16.77). Under the unadjusted dominant model, HOTAIR rs7958904 showed a non-significant protective effect (*p* = 0.077, OR = 0.46, 95% CI: 0.20–1.09). For CCAT1 rs6708563 in the additive model, a higher odds ratio was observed after adjustment for sex (*p* = 0.061, OR = 2.13, 95% CI: 0.97–4.70). The same effect emerged under the dominant model (*p* = 0.091, OR = 2.27, 95% CI: 0.88–5.87), suggesting a potential risk effect. Similarly, HOTAIR rs7958904 showed a non-significant protective effect in the additive model with sex interaction (*p* = 0.062, OR = 0.39, 95% CI: 0.15–1.05). Results with *p*-values lower than 0.091 are represented in Table 6; all others are represented in Supplementary Table S6.

### 3.5. Tumor Subtype-Specific Analyses (Colon vs. Rectal)

To examine the relationship between polymorphisms and tumor localization, we compared genotype frequencies between colon cancer and rectal cancer cases. The benign colon tumor cases were excluded from this analysis due to their low representation (*Nn* = 2).

#### 3.5.1. H19 rs2839698 Minor Allele (G) Favors Colon Cancer

**Figure 1:**
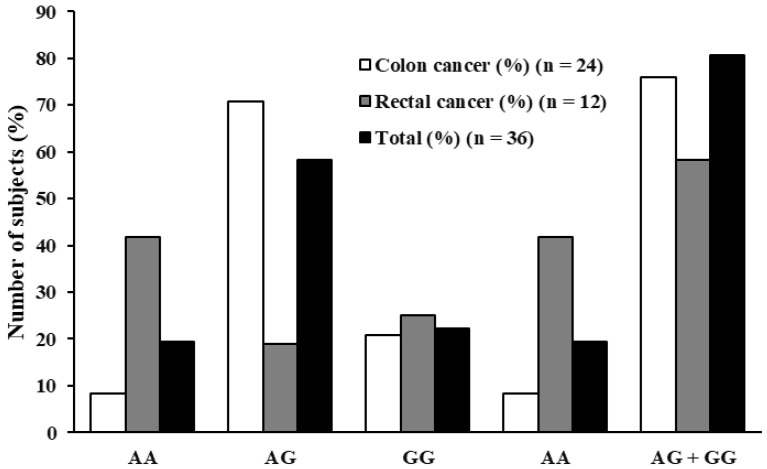
H19 rs2839698 genotype distribution in percentages. The first three columns correspond to the additive (AA, AG, GG) genetic model comparison, while the last two columns correspond to the dominant (AA, AG + GG) genetic model comparison.

A statistically significant difference was observed in genotype distribution between tumor subtypes in the case of H19 rs2839698. The minor allele (G) was more frequent in colon cancer cases compared to rectal cancer cases (*p* = 0.017 in the dominant model and *p* = 0.038 in the additive model (Figure 1 and Table 7)). In the full case group, which included both male and female participants, the presence of the minor allele (dominant model) was associated with a statistically significant reduction in the odds of rectal cancer (*p* = 0.029, unadjusted OR = 0.18, 95% CI: 0.02–0.81) (Table 8). This means that carriers of the minor allele had approximately 86% lower odds of having a rectal tumor compared to a colon tumor. After adjusting for sex, the effect remained significant (*p* = 0.040, adjusted OR = 0.14, 95% CI: 0.02–0.92) (Table 8). Among females, the dominant model showed no interpretable association, likely due to the small sample size. Among males, the direction of the association was consistent with the overall sample for the dominant model, although the result did not reach statistical significance. In this subgroup, OR of 0.32 was evaluated (*p* = 0.083, 95% CI: 0.09–1.16) (Table 8).

#### 3.5.2. PTCSC3 rs944289 Minor Allele (C) Favors Colon Cancer

**Figure 2:**
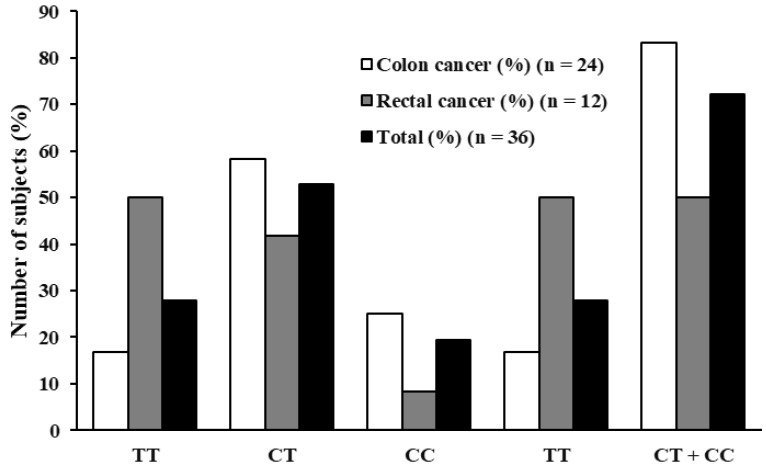
PTCSC3 rs944289 genotype distribution in percentages. The first three columns correspond to the additive (AA, AG, GG) genetic model comparison, while the last two columns correspond to the dominant (AA, AG + GG) genetic model comparison.

In the Chi-square test under the additive model, the overall difference in genotype frequencies between colon and rectal cancer patients was not statistically significant (*p* = 0.092) (Figure 2 and Table 9). However, a separate linear-by-linear association test indicated a significant linear trend across genotypes (χ^2^ = 4.179, *p* = 0.041), suggesting that an increasing number of minor alleles may be associated with a decreasing probability of rectal cancer. In the dominant model, a significant association was observed (χ^2^ = 4.431, *p* = 0.035) (Table 9). This result indicated that carriers of the minor allele (C) were more common among colon cancer cases than rectal cancer cases. To further quantify the observed effect and adjust for covariates, logistic regression analyses were conducted. In the full case group including both sexes, the dominant model showed a suggestive, protective association of the minor allele with rectal cancer (*p* = 0.043, unadjusted OR = 0.20, 95% CI: 0.04-0.95). (Table 10). This suggests that individuals carrying the minor allele had approximately 80% lower odds of having rectal cancer compared to colon cancer. In the additive model, a similarly suggestive protective effect emerged against rectal tumor localization (*p* = 0.048, unadjusted OR = 0.30, 95% CI: 0.09–0.99) (Table 10). However, after adjustment for sex, these associations were attenuated and no longer reached statistical significance. Sex-stratified analyses did not reveal statistically significant associations in either females or males (Supplementary Table S7). The effect was observed only in the combined case group.

#### 3.5.3. Lack of Subtype-Specific Associations Between Colon and Rectal Tumors in the Case of H19 rs2839698 and PTCSC3 rs944289

To determine whether an lncRNA variant is associated with a specific tumor subtype or just differentiates between tumor and non-tumor state in general, we conducted separate analyses in colon cancer vs. control and rectal cancer vs. control groups with the previously found significant variants. H19 rs2839698 variant showed a statistically significant association under the dominant model (*p* = 0.029, unadjusted OR = 0.18, 95% CI: 0.02–0.81) (Table 8), indicating a substantially lower frequency of the minor allele among rectal tumor cases compared to colon tumors. However, in the comparisons of either rectal vs. control or colon vs. control, no significant associations were observed. For PTCSC3 rs944289, the dominant and additive model revealed suggestive associations in the rectal vs. colon comparison (*p* = 0.043, unadjusted OR = 0.20, 95% CI: 0.04–0.95 and *p* = 0.048, unadjusted OR = 0.30, 95% CI: 0.09–0.99, respectively) (Table 10), with the minor allele being more frequent in colon tumors. In contrast, neither rectal vs. control nor colon vs. control comparisons showed significant associations (Supplementary Table S8).

### 3.6. Multiple Testing Correction

To control for multiple testing, we applied the Bonferroni correction. The significance threshold was adjusted by dividing α = 0.05 by the total number of primary tests (number of SNPs * number of genetic models * number of adjustments or interaction tests). Given the analysis of six SNPs under three genetic models and three types of adjustments (unadjusted, adjusted for sex, and sex interaction), this resulted in a corrected significance threshold of 0.05/54 = 0.00093. Several SNPs showed nominal associations with disease risk; however, none of the associations remained statistically significant after Bonferroni correction.

### 3.7. Post Hoc Power Analysis

The analysis showed that the study had limited power (~16–48%) to detect small-to-moderate genetic effects (Cohen’s w = 0.1–0.2), with an average MAF of 0.38. However, the study achieved acceptable power (~82%) to detect moderate-to-large effects (Cohen’s w = 0.3), corresponding approximately to an odds ratio of 1.8–2.0.

## 4. Discussion

In this exploratory genetic association study, we investigated six lncRNA-associated SNPs in relation to colorectal lesion susceptibility, including tumor subtype specificity and sex-dependent effects. Despite the limited sample size, several nominally significant associations emerged, primarily implicating variants within HOTAIR, H19, and PTCSC3 lncRNA genes; however, after Bonferroni correction, none of them remained significant. Although the overall analyses did not reveal any association in genotype or allele frequencies between cases and controls, the genotype frequency comparison revealed suggestive effects for HOTAIR rs7958904 and rs12826786 variants. However, logistic regression identified more specific patterns. Several additional findings demonstrated indicative associations. In our study, CCAT1 rs6708563 and HOTAIR rs7958904 showed a tendency toward increased risk and protective effects, respectively. Similarly, interaction models suggested a possible modifying role of sex for HOTAIR rs12826786 and PTCSC3 rs944289. As these results did not reach nominal significance, these observations should be interpreted more cautiously.

This study offers preliminary insights but has some limitations. First, the relatively small sample size (*n* = 91) reduces statistical power and precludes more detailed subgroup analyses, which may increase the risk of Type II errors for non-significant SNPs. Second, the case group includes both colorectal cancer and benign colon tumors, which introduce clinical heterogeneity and may dilute potential associations specific to malignant disease. Third, the lack of replication in an independent cohort and the potential population-specific effects may limit the generalizability of our findings. These factors should be considered when interpreting the results and highlight the need for validation in larger, diverse populations.

Previous studies of the HOTAIR rs12826786 polymorphism show heterogeneous results across cancer types and populations. In gastric cardia adenocarcinoma, Guo et al. linked the T allele to higher risk, advanced TNM stage, and increased HOTAIR expression predicting poor survival in Chinese patients [41]. A meta-analysis by Li et al. of Turkish, Iranian, and Chinese cohorts also associated the T allele and TT genotype with increased risk [42], whereas no effect was seen in gastric cancer in a Turkish population [43]. In contrast, in a Saudi CRC cohort Alzeer et al. reported a significant protective association of the TT genotype, particularly in male patients and in tumors located in the colon [44]. Our Hungarian data are consistent with this observation, with the T allele showing a possible protective association with CRC in additive and dominant models (*p* = 0.022 and *p* = 0.033, respectively).

The impact of the HOTAIR rs7958904 polymorphism on cancer risk appears to be strongly dependent on cancer type. In Chinese cohorts, several studies reported that the C allele or CC genotype increased susceptibility to cervical and breast cancer [45,46]. Akther et al. found similar associations in Bangladeshi women with cervical cancer [47]. In contrast, Wu et al. showed that the C allele decreased epithelial ovarian cancer risk [48], and Zhou et al. identified a protective effect against osteosarcoma in a Chinese cohort [49]. For gastric cancer, the C allele was linked to 1.5-fold increased risk in an Iranian cohort [50]. Results in CRC are inconsistent as Kim et al. reported that CC genotype predicted poorer prognosis and higher cancer-related mortality in a Korean cohort [51], whereas Xue et al. demonstrated a protective association of the C allele particularly in older individuals, women, and non-smokers [52]. A meta-analysis of predominantly Asian populations showed an overall protective effect in gastric and colorectal cancer [53]. Consistent with these latter findings, our data revealed a possible protective effect of the rs7958904 C allele in the dominant model (*p* = 0.043) in females, suggesting rs7958904 may reduce colorectal lesion susceptibility in a sex-dependent manner.

Earlier studies have demonstrated that the H19 rs2839698 A allele increases susceptibility to colorectal and other digestive cancers. In a large Chinese cohort, Li et al. reported higher CRC risk for A allele carriers (OR = 1.20, 95% CI: 1.05–1.36), especially in colon tumors, well-differentiated histology, and advanced Duke’s stage [54]. Meta-analyses confirmed digestive cancer risk associations under several genetic models [55,56,57]. Wu et al. found similar links for hepatocellular carcinoma in Chinese patients [58], whereas Verhaegh et al. reported a protective effect in bladder cancer [59]. In our Hungarian cohort, rs2839698 was not associated with overall colorectal lesion risk; instead, the G allele was more frequent in colon tumors and conferred a potential reduced risk of rectal cancer (*p* = 0.029, OR = 0.18), suggesting that rs2839698 may influence tumor subsite rather than general colorectal lesion susceptibility.

Genome-wide association studies identified rs944289 as a susceptibility locus for papillary thyroid carcinoma (PTC). Jendrzejewski et al. showed that this variant modulates PTCSC3 expression, a thyroid-specific tumor suppressor strongly downregulated in tumors, especially in T allele carriers [60], and meta-analyses confirmed its association with PTC risk [61,62,63,64]. Although most research has focused on thyroid cancer, rs944289 has also been examined in digestive system cancers. In a Chinese cohort, Cao et al. found no overall association with esophagogastric junction adenocarcinoma (EGJA), but stratified analyses showed a protective CT genotype in individuals under 60 years and increased risk for the TT genotype among smokers [65]. In another Chinese study, Wang et al. reported a decreased CRC risk for the TT genotype overall and in subgroups, yet tumor site analyses linked TT genotype to increased rectal and colon cancer risk [37], indicating context- and population-dependent effects. In contrast, our data showed that the C allele (the minor allele in European populations) was associated with lower rectal cancer risk but was more frequent among colon cancer cases, suggesting a possible one-sided effect favoring colon tumor development.

Previous work supports a functional role for HOTAIR rs12826786: in glioma tissue, the CT genotype shows higher HOTAIR expression than TT, indicating a cis-eQTL-like effect [66]. This variant is not a significant eQTL in GTEx v8 normal tissues [67], suggesting a tissue-specific regulatory action. For rs7958904, rs2839698, and rs944289, we found no published CRC-specific eQTLs. However, rs7958904 may alter HOTAIR’s secondary structure (in silico CRC-related study [52]), H19 rs2839698 is linked to higher risk in digestive system cancers [68], and PTCSC3 rs944289 reduces promoter activity and PTCSC3 expression in thyroid carcinoma [60] (GWAS Catalog: GCST000335). These findings imply tissue- or context-specific regulation, and the absence of CRC-specific eQTL data likely reflects limited datasets rather than lack of effect, supporting the biological plausibility of our CRC association.

It should be noted that allele frequencies, including which allele is considered the minor allele, may differ substantially between populations. Such differences could partly account for the discrepancies observed between our findings in a European cohort and those reported in Asian populations. Collectively, these observations suggest that lncRNA-associated polymorphisms may exert cancer type and sex-specific effects rather than universal effects across patients with colorectal lesions and controls. While most studies examined these SNPs in larger and mainly Asian cohorts, our findings expand this work with an additional Central European population and specifically with colorectal lesions. The results discussed here imply that the same lncRNA variants could contribute to tumorigenesis differently depending on tissue context, sex, and population-specific genotype or allele frequencies. In this context, the results of the post hoc power analysis provide an important perspective, as the absence of statistically significant associations for some SNPs may reflect the study’s limited power to detect weaker effects rather than a true lack of biological relevance. Despite the small sample size, this cohort represents a less frequent and genetically homogeneous group of Hungarian patients. Therefore, as this was an exploratory phase, we plan a validation phase with a larger patient cohort based on these results.

## 5. Conclusions

To our best knowledge, this is the first study to investigate these polymorphisms in a Hungarian colorectal lesion population. These preliminary results suggest that specific lncRNA polymorphisms may contribute to colorectal lesion risk or may highlight differences in tumor localization or sex, emphasizing the importance of integrating genetic-, molecular-, and population-level factors when assessing lncRNA variants as biomarkers. Future directions include validating our findings in larger, multicenter Hungarian and Central European cohorts to enhance statistical power and applicability.

## Figures and Tables

**Table 1:** Rationale for SNP selection for this study.

SNP	lncRNA	Genomic Region	Functional/Biological Relevance	Rationale for Inclusion
rs6708563	CCAT1	8q24.21	CCAT1 promotes tumorigenesis through MYC activation and WNT/β-catenin signaling; overexpressed in CRC tissue [23,24]	A functional SNP within CCAT1 located in the 8q24 cancer susceptibility region, potentially influencing lncRNA expression and implicated in colorectal carcinogenesis.
rs6983267	CCAT2	8q24.21	CCAT2 enhances MYC expression, drives WNT/β-catenin pathway activation; upregulated in CRC tissues [24]	A high-confidence CRC susceptibility SNP at the most widely replicated 8q24 risk locus with strong biological and epidemiological support.
rs2839698	H19	11p15.5	H19 overexpressed in CRC serum and tumor tissue; linked to invasion, metastasis, and poor prognosis [25,26,27]	A functionally relevant H19 variant linked to altered expression and cancer risk, implicated in CRC progression.
rs7958904	HOTAIR	12q13	HOTAIR drives epigenetic silencing of tumor suppressor genes; associated with advanced CRC and poor survival [28,29,30,31,32,33]	An HOTAIR variant affecting expression and cancer susceptibility in a key metastasis-related lncRNA with strong prognostic relevance.
rs12826786	HOTAIR	12q13	Same biological role as above; HOTAIR is a key epigenetic regulator in CRC	An HOTAIR variant implicated in modulating lncRNA activity and cancer risk, complementing rs7958904 by capturing additional regulatory variation.
rs944289	PTCSC3	9q22	PTCSC3 acts as a tumor-suppressive lncRNA; with a role in many types of cancer [34,35,36,37,38]	A PTCSC3 variant associated with altered expression and cancer susceptibility, providing a possible insight into genetic variation in a tumor-suppressive lncRNA that might be relevant to CRC.

**Table 2:** Clinical and demographic characteristics of the study population.

	Control (%)	Case (%)	Total
Female	33 (62.3)	21 (55.3)	54
Male	20 (37.7)	17 (44.7)	37
Total	53 (100)	38 (100)	91
Age (years, median)	60	71.5	

**Table 3:** Diagnosis groups of case population.

Diagnosis	Case (%)
Colon cancer	24 (63.1)
Rectal cancer	12 (31.6)
Benign colon tumor	2 (5.3)
Total	38 (100)

**Table 4:** Genotype distribution in additive, dominant, and recessive models for all examined SNPs and MAF results for this study’s control population with reference values of 1000 Genomes (European) and gnomAD (European, non-Finnish) databases. NA = not available.

Model	Genotype	Control (%) (*n* = 53)	Case (%) (*n* = 38)	Total (%) (*n* = 91)	MAF (%) (Control)	MAF (%) (1000 Genomes)	MAF (%) (GnomAD)
H19 rs2839698							
Additive	AA	13 (24.5)	7 (18.4)	20 (22.0)	51.8	48.8	NA
	AG	25 (47.2)	22 (57.9)	47 (51.6)			
	GG	15 (28.3)	9 (23.7)	24 (26.4)			
Dominant	AA	13 (24.5)	7 (18.4)	20 (22.0)			
	AG + GG	40 (75.5)	31 (81.6)	71 (78.0)			
Recessive	AA + AG	38 (71.7)	29 (76.3)	67 (73.6)			
	GG	15 (28.3)	9 (23.7)	24 (26.4)			
CCAT1 rs6708563							
Additive	CC	37 (69.8)	21 (55.3)	58 (63.7)	16.0	30.7	32.5
	CT	15 (28.3)	14 (36.8)	29 (31.9)			
	TT	1 (1.9)	3 (7.9)	4 (4.4)			
Dominant	CC	37 (69.8)	21 (55.3)	58 (63.7)			
	CT + TT	16 (30.2)	17 (44.7)	33 (36.3)			
Recessive	CC + CT	52 (98.1)	35 (92.1)	87 (95.6)			
	TT	1 (1.9)	3 (7.9)	4 (4.4)			
CCAT2 rs6983267							
Additive	TT	16 (30.2)	11 (28.9)	27 (29.7)	46.2	49.9	49.1
	GT	25 (47.2)	14 (36.8)	39 (42.9)			
	GG	12 (22.6)	13 (34.2)	25 (27.5)			
Dominant	TT	16 (30.2)	11 (28.9)	27 (29.7)			
	GT + GG	37 (69.8)	27 (71.1)	64 (70.3)			
Recessive	TT + GT	41 (77.4)	25 (65.8)	66 (72.5)			
	GG	12 (22.6)	13 (34.2)	25 (27.5)			
HOTAIR rs7958904							
Additive	GG	18 (34.0)	20 (52.6)	38 (41.8)	37.7	30.3	46.9
	CG	30 (56.6)	14 (36.8)	44 (48.4)			
	CC	5 (9.4)	4 (10.5)	9 (9.9)			
Dominant	GG	18 (34.0)	20 (52.6)	38 (41.8)			
	CG + CC	35 (66.0)	18 (47.4)	53 (58.2)			
Recessive	GG +CG	48 (90.6)	34 (89.5)	82 (90.1)			
	CC	5 (9.4)	4 (10.5)	9 (9.9)			
HOTAIR rs12826786							
Additive	CC	20 (37.7)	21 (55.3)	41 (45.1)	35.8	29.5	37.5
	CT	28 (52.8)	14 (36.8)	42 (46.2)			
	TT	5 (9.4)	3 (7.9)	8 (8.8)			
Dominant	CC	20 (37.7)	21 (55.3)	41 (45.1)			
	CT + TT	33 (62.3)	17 (44.7)	50 (54.9)			
Recessive	CC + CT	48 (90.6)	35 (92.1)	83 (91.2)			
	TT	5 (9.4)	3 (7.9)	8 (8.8)			
PTCSC3 rs944289							
Additive	TT	19 (35.8)	11 (28.9)	30 (33.0)	43.4	41.9	41.5
	CT	22 (41.5)	20 (52.6)	42 (46.2)			
	CC	12 (22.6)	7 (18.4)	19 (20.9)			
Dominant	TT	19 (35.8)	11 (28.9)	30 (33.0)			
	CT + CC	34 (64.2)	27 (71.1)	61 (67.0)			
Recessive	TT + CT	41 (77.4)	31 (81.6)	72 (79.1)			
	CC	12 (22.6)	7 (18.4)	19 (20.9)			

**Table 5:** Hardy–Weinberg equilibrium test results for this study’s control population with Chi-square test and *p*-values.

SNP	Minor Allele	χ^2^	*p*-Value
H19 rs2839698	G	0.162	0.687
CCAT1 rs6708563	T	0.137	0.711
CCAT2 rs6983267	G	0.139	0.709
HOTAIR rs7958904	C	2.217	0.136
HOTAIR rs12826786	T	1.17	0.279
PTCSC3 rs944289	C	1.275	0.259

**Table 6:** Results of logistic regression analyses on different genetic models (additive, dominant). Unadjusted: including only the SNP. Adjusted for sex: including the SNP and sex as a covariate. Sex interaction (men vs. women): SNP × sex interaction, indicating how the SNP effect differs between males and females. Sex interaction (main effect, reference sex = female): represents the odds ratio for the SNP in the reference sex (female) estimated from a model including the SNP, sex, and the SNP × sex interaction term. * marks significant *p*-values.

SNP	Model	OR	95% CI Lower	95% CI Upper	*p*-Value
CCAT1 rs6708563	additive, adjusted for sex	2.13	0.97	4.70	0.061
CCAT1 rs6708563	dominant, adjusted for sex	2.27	0.88	5.87	0.091
HOTAIR rs7958904	additive, sex interaction (main effect, reference sex = female)	0.39	0.15	1.05	0.062
HOTAIR rs7958904	dominant, sex interaction (main effect, reference sex = female)	0.31	0.10	0.96	0.043 *
HOTAIR rs7958904	dominant, unadjusted	0.46	0.20	1.09	0.077
HOTAIR rs12826786	additive, sex interaction (main effect, reference sex = female)	0.29	0.10	0.84	0.022 *
HOTAIR rs12826786	additive, sex interaction (men vs. women)	4.01	0.96	16.77	0.057
HOTAIR rs12826786	dominant, sex interaction (main effect, reference sex = female)	0.29	0.09	0.90	0.033 *
PTCSC3 rs944289	additive, sex interaction (men vs. women)	2.88	0.86	9.71	0.089

**Table 7:** H19 rs2839698 genotype distribution in the additive and dominant genetic models with χ^2^ and *p*-values. * marks significant *p*-values.

Model	Genotype	Colon Cancer (%) (*n* = 24)	Rectal Cancer (%) (*n* = 12)	Total (%) (*n* = 36)	χ^2^	*p*-Value
Additive	AA	2 (8.3)	5 (41.7)	7 (19.4)	6.563	0.038 *
	AG	17 (70.8)	4 (19)	21 (58.3)		
	GG	5 (20.8)	3 (25)	8 (22.2)		
Dominant	AA	2 (8.3)	5 (41.7)	7 (19.4)	5.675	0.017 *
	AG + GG	22 (75.9)	7 (58.3)	29 (80.6)		

**Table 8:** Logistic regression results for H19 rs2839698 in the dominant genetic model. * marks significant *p*-values.

SNP	Model	OR	95% CI Lower	95% CI Upper	*p*-Value
H19 rs2839698	dominant, unadjusted	0.18	0.02	0.81	0.029 *
H19 rs2839698	dominant, adjusted for sex	0.14	0.02	0.92	0.040 *
H19 rs2839698	dominant, stratified for males	0.32	0.09	1.16	0.083

**Table 9:** PTCSC3 rs944289 genotype distribution in the additive and dominant genetic models with χ^2^ and *p*-values. * marks significant *p*-values.

Model	Genotype	Colon Cancer (%) (*n* = 24)	Rectal Cancer (%) (*n* = 12)	Total (%) (*n* = 36)	χ^2^	*p*-Value
Additive	TT	4 (16.7)	6 (50)	10 (27.8)	4.764	0.092
	CT	14 (58.3)	5 (41.7)	19 (52.8)		
	CC	6 (25.0)	1 (8.3)	7 (19.4)		
Dominant	TT	4 (16.7)	6 (50)	10 (27.8)	4.431	0.035 *
	CT + CC	20 (83.3)	6 (50)	26 (72.2)		

**Table 10:** Logistic regression results for PTCSC3 rs944289 in the dominant and additive genetic models. * marks significant *p*-values.

SNP	Model	OR	95% CI Lower	95% CI Upper	*p*-Value
PTCSC3 rs944289	dominant, unadjusted	0.20	0.04	0.95	0.043 *
PTCSC3 rs944289	dominant, adjusted for sex	0.20	0.04	1.00	0.050
PTCSC3 rs944289	additive, unadjusted	0.30	0.09	0.99	0.048 *
PTCSC3 rs944289	additive, adjusted for sex	0.31	0.09	1.04	0.057

## Data Availability

The original contributions presented in this study are included in the article and in the Supplementary Materials. Further inquiries can be directed to the corresponding authors.

## Supplementary Materials

The following supporting information can be downloaded at: https://www.mdpi.com/article/10.3390/biomedicines13123058/s1, Table S1: Genotype data of study participants for each SNP; Table S2: HWE test results; Table S3: Genotype distribution for the six SNP under dominant, recessive, and additive models with χ2 *p*-values; Table S4: Fisher’s two-sided *p*-values for the six SNP under dominant and recessive models; Table S5: Allele frequency counts and Pearson χ^2^ *p*-values; Table S6: Logistic regression results; Table S7: Logistic regression results for PTCSC3 rs944289 in the dominant and additive genetic models, with sex-stratified analyses; Table S8: Logistic regression results for rectal vs. control and colon vs. control comparisons on selected SNPs.

## Abbreviations

The following abbreviations are used in this manuscript:

CRC	Colorectal cancer
lncRNA	Long non-coding RNA
SNP	Single-nucleotide polymorphisms
HOTAIR	HOX transcript antisense RNA
PTCSC3	Papillary thyroid carcinoma susceptibility candidate 3
CCAT1	Colon cancer-associated transcript 1
CCAT2	Colon cancer-associated transcript 2
qPCR	Quantitative polymerase chain reaction
FDR	False discovery rate
ASR	Age-standardized incidence rate
HWE	Hardy–Weinberg equilibrium
SD	Standard deviation
TNM	Tumor–node–metastasis
PTC	Papillary thyroid carcinoma
EGJA	Esophagogastric junction adenocarcinoma
